# Impact of positive biphasic pressure during low and high inspiratory efforts in *Pseudomonas aeruginosa*-induced pneumonia

**DOI:** 10.1371/journal.pone.0246891

**Published:** 2021-02-12

**Authors:** Daniela G. da Cruz, Raquel F. de Magalhães, Gisele A. Padilha, Mariana C. da Silva, Cassia L. Braga, Adriana R. Silva, Cassiano F. Gonçalves de Albuquerque, Vera L. Capelozzi, Cynthia S. Samary, Paolo Pelosi, Patricia R. M. Rocco, Pedro L. Silva

**Affiliations:** 1 Laboratory of Pulmonary Investigation, Carlos Chagas Filho Institute of Biophysics, Federal University of Rio de Janeiro, Rio de Janeiro, Rio de Janeiro, Brazil; 2 Laboratory of Immunopharmacology, Fundação Oswaldo Cruz, Instituto Oswaldo Cruz, Rio de Janeiro, Brazil; 3 Department of Pathology, School of Medicine, University of São Paulo, São Paulo, Brazil; 4 Anesthesiology and Intensive Care, San Martino Policlinico Hospital, IRCCS for Oncology and Neurosciences, Genoa, Italy; 5 Department of Surgical Sciences and Integrated Diagnostics, University of Genoa, Genoa, Italy; Emory University School of Medicine, UNITED STATES

## Abstract

**Background:**

During pneumonia, normal alveolar areas coexist adjacently with consolidated areas, and high inspiratory efforts may predispose to lung damage. To date, no study has evaluated different degrees of effort during Biphasic positive airway pressure (BIVENT) on lung and diaphragm damage in experimental pneumonia, though largely used in clinical setting. We aimed to evaluate lung damage, genes associated with ventilator-induced lung injury (VILI) and diaphragmatic injury, and blood bacteria in pressure-support ventilation (PSV), BIVENT with low and high inspiratory efforts in experimental pneumonia.

**Material and methods:**

Twenty-eight male Wistar rats (mean ± SD weight, 333±78g) were submitted *Pseudomonas aeruginosa*-induced pneumonia. After 24-h, animals were ventilated for 1h in: 1) PSV; 2) BIVENT with low (BIVENT_Low-Effort_); and 3) BIVENT with high inspiratory effort (BIVENT_High-Effort_). BIVENT was set at P_high_ to achieve V_T_ = 6 ml/kg and P_low_ at 5 cmH_2_O (n = 7/group). High- and low-effort conditions were obtained through anaesthetic infusion modulation based on neuromuscular drive (P_0.1_). Lung mechanics, histological damage score, blood bacteria, and expression of genes related to VILI in lung tissue, and inflammation in diaphragm tissue.

**Results:**

Transpulmonary peak pressure and histological damage score were higher in BIVENT_High-Effort_ compared to BIVENT_Low-Effort_ and PSV [16.1 ± 1.9cmH_2_O *vs* 12.8 ± 1.5cmH_2_O and 12.5 ± 1.6cmH_2_O, p = 0.015, and p = 0.010; median (interquartile range) 11 (9–13) *vs* 7 (6–9) and 7 (6–9), p = 0.021, and p = 0.029, respectively]. BIVENT_High-Effort_ increased interleukin-6 expression compared to BIVENT_Low-Effort_ (p = 0.035) as well as expressions of cytokine-induced neutrophil chemoattractant-1, amphiregulin, and type III procollagen compared to PSV (p = 0.001, p = 0.001, p = 0.004, respectively). Tumour necrosis factor-α expression in diaphragm tissue and blood bacteria were higher in BIVENT_High-Effort_ than BIVENT_Low-Effort_ (p = 0.002, p = 0.009, respectively).

**Conclusion:**

BIVENT requires careful control of inspiratory effort to avoid lung and diaphragm damage, as well as blood bacteria. P_0.1_ might be considered a helpful parameter to optimize inspiratory effort.

## Introduction

Pneumonia caused by *Pseudomonas aeruginosa* is frequent in intensive care unit patients [1] and can disrupt upper and lower airway homeostasis by damaging the epithelium and evading innate and adaptive immune responses [2]. Consequently, there is a widespread focal consolidation, which may lead to heterogeneous distortion of lung parenchyma during mechanical ventilation. Partial ventilatory support allows spontaneous breathing during mechanical ventilation, leading to a reduction in the need for sedation [3], improvement in hemodynamic [4], and maintenance of respiratory muscle activity [5]. Biphasic positive airway pressure (BIVENT) is a partial ventilatory support mode that employs pressure-controlled, time-cycled ventilation set at two levels of continuous positive airway pressure (CPAP) with unrestricted spontaneous breathing during the ventilatory cycle. In experimental acute respiratory distress syndrome (ARDS), BIVENT has shown advantages over controlled mechanical ventilation, reducing the risk of ventilator-induced lung injury (VILI) [6–8]. Additionally, lung damage and worse outcomes might occur during airway pressure release ventilation (APRV) [9] when high inspiratory effort is present [10–12]. On the other hand, recent preclinical [13] and clinical studies [14] have shown that increased effort during partial ventilatory support is not associated with worse outcomes in early ARDS. Nevertheless, in pneumonia, where normal alveolar areas coexist adjacently with consolidated areas, high inspiratory efforts may predispose to lung damage. To date, no study has evaluated the impact of different degrees of effort during BIVENT on lung and diaphragm damage in experimental pneumonia. We hypothesized that BIVENT may promote beneficial effects only with low inspiratory effort. For this purpose, lung damage, biological markers associated with both ventilator-induced lung injury (VILI) and diaphragmatic injury, and bacterial translocation were evaluated in three ventilation strategies—BIVENT with low inspiratory effort, BIVENT with high inspiratory effort, and pressure-support ventilation (PSV)—in a model of *Pseudomonas aeruginosa*-induced pneumonia.

## Methods

### Study approval

This study was approved by the Ethics Committee of the Health Science Centre (CEUA 116/16), Federal University of Rio de Janeiro, Rio de Janeiro, Brazil. All animals received humane care in compliance with the “Principles of Laboratory Animal Care” formulated by the National Society for Medical Research and the Guide for the Care and Use of Laboratory Animals prepared by the National Academy of Sciences, USA. The present study followed the ARRIVE guidelines for reporting of animal research [15]. Animals were housed at a controlled temperature (23°C) and controlled light–dark cycle (12–12 h), with free access to water and food.

### Bacterial preparation

*Pseudomonas aeruginosa* (ATCC27853), supplied by the FIOCRUZ Bacterial Culture Collection, was cultured overnight in Luria Broth Base (Invitrogen^™^ by Life Technologies, Carlsbad, CA, USA) at 37°C to obtain stationary-phase microorganisms. Subsequently, the culture medium containing the bacteria was centrifuged at 6,500 g for 15 minutes at 4°C and the pellet was washed and resuspended in sterile saline. The sample was analysed by spectrophotometry and adjusted to the desired dose of 5×10^7^ colony-forming units (CFUs) [16]. In previous studies, this level of blood bacteria resulted in pneumonia with no mortality [16].

### Pneumonia induction

In the early morning (8:00 a.m.), 28 male Wistar rats (mean body weight ± SD, 333±78g) were anesthetized under spontaneous breathing with 1.5–2.0% isoflurane (Isoforine^®^; Cristália, Itapira, SP, Brazil) and underwent intratracheal instillation of the above-mentioned bacterial cultures. This bacterial strain is deposited in the Culture Collection of Hospital-Acquired Bacteria (CCBH) located at the Hospital Infection Research Laboratory at Oswaldo Cruz Institute, FIOCRUZ, Brazil.

### Animal preparation and experimental protocol

**Fig 1:**
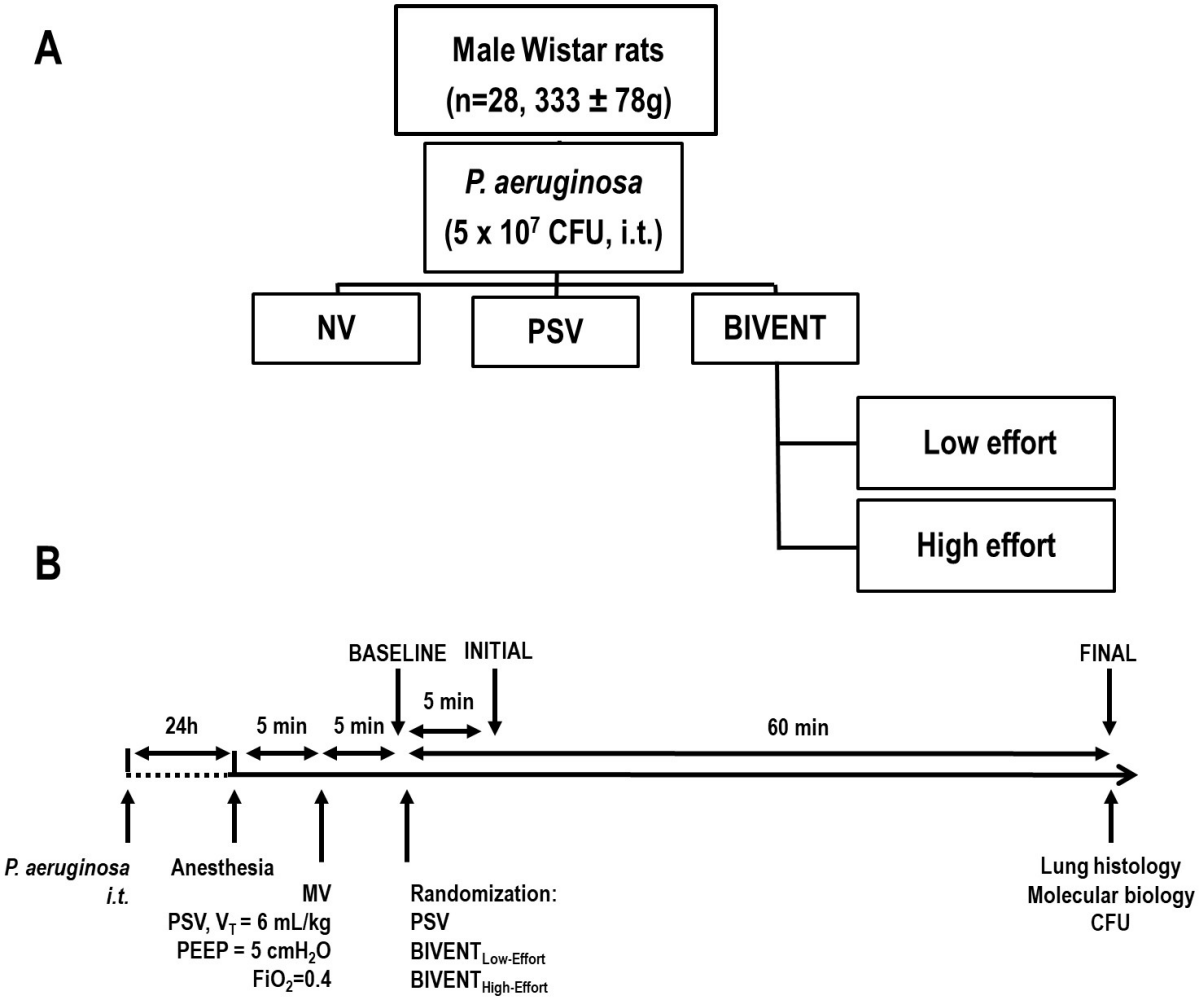
A. Experimental design. i.t.: intratracheal; NV: non-ventilated; PSV: pressure-support ventilation; BIVENT: biphasic positive airway pressure ventilation; Low effort: anaesthesia infusion was maintained to keep inspiratory effort low, according to oesophageal pressure decay; High effort: anaesthesia infusion was reduced to achieve high inspiratory effort according to oesophageal pressure decay (n = 7/group). B. Timeline of the experiments. i.t.: intratracheal; CFU: colony-forming unit; MV: mechanical ventilation; V_T_: tidal volume; FiO_2_: inspiratory fraction of oxygen; PEEP: positive end-expiratory pressure; CFU: colony-forming unit.

After 24 h, animals were premedicated intraperitoneally (i.p.) with midazolam (1–2 mg/kg) and anesthetized with ketamine (100 mg/kg, i.p.). An intravenous (i.v.) catheter (Jelco 24G, Becton, Dickinson and Company, New Jersey, NJ, USA) was inserted into the tail vein, and anaesthesia induced and maintained with midazolam (2 mg/kg/h) and ketamine (50 mg/kg/h). The adequacy of anaesthesia was assessed by response to a nociceptive stimulus before surgery. Body temperature was maintained at 37.5 ± 1°C with a heating bed (EFF 421, INSIGHT^®^, Brazil). The neck area was anesthetized by subcutaneous injection of 0.4 ml lidocaine (2%), a tracheostomy was performed, and a polyethylene cannula (internal diameter 1.8 mm, length 7.5 cm; PE 240, Intramedic^®^, Clay-Adams Inc., New York, USA) was advanced into the trachea. A second catheter (18G; Arrow International, USA) was then placed in the right internal carotid artery for blood sampling and gas analysis (Radiometer ABL80 FLEX, Copenhagen NV, Denmark), as well as monitoring of mean arterial pressure (MAP) (Networked Multiparameter Veterinary Monitor LifeWindow 6000 V; Digicare Animal Health, Boynton Beach, FL, USA). Animals were connected to an airway pressure transducer (UT-PDP-70; SCIREQ, Canada) and a two-sidearm pneumotachograph (internal diameter 2.7 mm, length 25.7 mm, internal volume 0.147 ml, airflow resistance 0.0057 cm H_2_O·ml^-1^·s^-1^) [17] connected to a differential pressure transducer (UT-PDP-02, SCIREQ, Montreal, QC, Canada), for airflow (V′) measurement. A 30-cm-long water-filled catheter (PE-205; Becton, Dickinson and Company) with side holes at the tip, connected to a differential pressure transducer (UT-PL-400; SCIREQ, Canada), was used to measure the oesophageal pressure, and proper positioning was assessed using the “occlusion test” as described elsewhere [18]. Seven of the 28 rats were subjected to *P*. *aeruginosa* instillation, but not ventilated. This non-ventilated (NV) group was used for molecular biology analysis. Twenty-one rats were mechanically ventilated (SERVO-i; MAQUET, Solna, Sweden) in PSV with ΔP set to achieve a tidal volume (V_T_) of 6 ml/kg, PEEP of 5 cmH_2_O, and FiO_2_ equal to 0.4. Flow trigger sensitivity was adjusted at BASELINE for adequate inspiratory effort, according to esophageal pressure (P_es_) decay. No additional changes to flow trigger sensitivity were done at any point in the experiment. Shortly after, animals were randomly assigned to: 1) PSV according to previous ventilatory settings; 2) BIVENT_Low-Effort_; or 3) BIVENT_High-Effort_ (n = 7/group) (Fig 1A). In PSV and BIVENT_Low-Effort_, anaesthesia was titrated to obtain low effort according to P_es_ decay, while in BIVENT_High-Effort_ the anaesthesia infusion was reduced to reach high effort according to P_es_ decay. The criteria to observe P_es_ decay was based on neuromuscular drive (P_0.1_ level). Anaesthesia titration lasted few minutes, without affecting the total time under mechanical ventilation among groups. For BIVENT, the following parameters were adjusted: airway pressure spent during inspiratory time (P_high_) to reach V_T_ = 6 ml/kg, P_low_ = 5 cmH_2_O, and inspiratory time (T_high_) = 0.3 seconds. Blood gas analysis (Radiometer, Copenhagen, Denmark) and mechanical data were obtained at INITIAL and at the end of 1 h of mechanical ventilation (FINAL) under FiO_2_ = 0.4 (Fig 1B). At FINAL, blood samples were taken for measurement of bacterial load (CFU count). Animals were euthanized by intravenous overdose of sodium thiopental (60 mg/kg; Cristália, Brazil). The left lung was removed for quantification of heterogeneous airspace enlargement and pneumonia score, and the right lung and diaphragm were harvested for gene expression analysis by quantitative real-time reverse transcription polymerase chain reaction (RT-PCR).

### Data acquisition and respiratory system mechanics

Airflow, airway pressure (P_aw_), and P_es_ were recorded continuously throughout the experiments by a computer running customer-made software written in LabVIEW (National Instruments, USA). V_T_ was calculated by digital integration of the airflow signal. The total respiratory rate (RR) was calculated from the P_es_ swings as the frequency per minute of each type of breathing cycle. Peak transpulmonary pressure (Ppeak,_L_) was calculated as the difference between tracheal and esophageal pressure. P_0.1_ is the esophageal pressure measured 100 ms after the onset of inspiratory effort, and it reflects the neuromuscular drive. All signals were amplified in a three-channel signal conditioner (TAM-DHSE Plugsys Transducers Amplifiers, Module Type 705/2, Harvard Apparatus, Holliston, Massachusetts, USA) and sampled at 200 Hz with a 12-bit analog-to-digital converter (National Instruments; Austin, Texas, USA) [19]. All mechanical data were computed offline by a routine written in MATLAB (Version R2007a; The Mathworks Inc., USA).

### Histology

#### Pneumonia score

The left lung was removed, fixed, and embedded in paraffin. The slides containing the lung sections were stained with haematoxylin and eosin and analysed according to their qualitative and quantitative aspects. For the descriptive analysis, the entire lamina surface was observed, with all pulmonary structures represented, under incremental magnification (25×, 100×, and 400×). *Pseudomonas aeruginosa*-induced lung damage was quantified by a pulmonary pathology specialist (V.L.C.) blinded to group assignment using a weighted scoring system as previously described [20, 21]. Assessment of perivascular oedema/haemorrhage, septal neutrophil and vasculitis features were based on a previous study that highlighted the histological characteristics most commonly found in models of pneumonia [22]. Values from 0 to 4 were used to represent the severity of a given characteristic, with 0 for no effect and 4 for maximum severity. The extent of involvement in each field of view was also scored on a scale from 0 to 4, with 0 for no appearance and 4 for full involvement. Results were calculated as the product of severity and extent of each feature, thus ranging from 0 to 16. Considering that the total lung damage score was the sum of three features, the pneumonia score ranged from 0 to 48.

### Immunohistochemistry analysis

For the immunohistochemistry analysis, the lung sections were subjected to a high temperature (121°C) for 1 minute for antigen retrieval. After blocking of nonspecific sites with 3% hydrogen peroxide for 5 minutes, the specimens were stained with rabbit polyclonal CINC-1 antibody (Millipore; 1:100 dilution, Temecula, California, USA). The reactions were stained with Vectastain ABC (Vector Laboratories). The colour was developed with 3,3-diaminobenzidinetetrahydrochloride (Vector Laboratories) and counterstained with H&E.

### Immunofluorescence and confocal analysis

To perform immunostaining for neutrophils and occludin, lung slides were mounted on 3-aminopropyltriethoxysilane (Sigma Chemical Co., St. Louis, MO, USA), dewaxed in xylene, and hydrated in graded ethanol. Antigen retrieval was accomplished using enzymatic treatment with pepsin from porcine gastric mucosa (10,000 dry units/mL) (Sigma Chemical Co. St. Louis, MO, USA) in acetic acid buffer at 0.5 N for 30 minutes at 37°C. Nonspecific sites were blocked with 5% bovine serum albumin (BSA) in phosphate-buffered saline (PBS) for 30 minutes, at room temperature. The lung specimens were incubated overnight at 4°C with rabbit monoclonal antibody to neutrophil elastase [clone EPR7479; 1:100; ABCAM; USA] and rabbit monoclonal antibody to occludin [clone EPR20992; 1:300; ABCAM, USA]. The lung sections were subsequently washed in PBS with Tween 20 0.05% and incubated for 60 minutes at room temperature. Bound antibodies were visualized with Alexa 488-conjugated anti-mouse IgG and Alexa 546-conjugated anti-rabbit IgG (Invitrogen, Eugene, OR, USA). Specimens were visualized in an immunofluorescence microscope (OLYMPUS BX51). For three-dimensional reconstruction by confocal microscopy, the same procedure described above was followed, but slides were viewed under a laser-scanning microscope (Zeiss LSM 510 META/UV, Germany).

Immunohistochemistry and immunofluorescence staining markers were scored using a membrane pattern to analyze occludin and a cytoplasmic pattern to analyze CINC-1. The intensity and extent of staining were scored as 0 (no staining), 1 (weak staining and focal extension), 2+ (moderate staining and focal extension), 3+ (moderate staining and diffuse extension), and 4+ (strong expression and diffuse extension). The total score was obtained by multiplying extension and intensity, yielding a final score of up to 16.

### Morphometric analysis

#### Quantification of heterogeneous airspace enlargement

Airspace enlargement was assessed by measuring the mean linear intercept between alveolar walls at a magnification of × 400, as described elsewhere [19, 23, 24]. To characterize the heterogeneity of airspace enlargement, the central moment of the mean linear intercept (D_2_ of mean linear intercept between alveolar walls) was computed from 20 airspace measurements [25], according to Eq 1:

$$D_{2}=\mu \cdot (1+\frac{\sigma^{2}}{\mu^{2}+\sigma^{2}})\cdot (2+\sigma \cdot \frac{\gamma }{\mu })$$ (1)

where μ is the mean, σ is the variance of airspace diameters, and γ is the skewness of the diameter distribution. After D_2_ calculation, the heterogeneity index (β) was derived from D_2_ and mean linear intercept between alveolar wall values by their ratio [19, 23, 24]. Quantification of heterogeneous airspace enlargement was performed to properly show heterogeneous airspace enlargement, lung slices were scanned (3DHISTECH^®^, Budapest, Hungary). All analyses were performed by one of the authors (M.C.S.), who was blinded to group assignment.

#### Blood bacterial load

Blood samples (20 μL) were seeded in Petri dishes with Tryptic Soy Agar growth medium (Fluka Analytical, St Louis, MO, USA). Manual CFU counts were performed after 24 h of incubation at 37°C.

#### Enzyme-linked immunosorbent assay of lung tissue

Protein levels of CINC-1 (1009127, Murine KC, Preprotech, United States) in lung tissue were measured by enzyme-linked immunosorbent assay (ELISA). The sample was homogenized in lysis buffer (1 M HEPEs, 0.5 M EDTA, 1 M sucrose, 200 mM NaF, Roche protease inhibitor cocktail) and total protein was quantified by Bradford’s assay (5000205, Quick Start^™^ Bradford 1× Dye Reagent). ELISA was performed as per manufacturer instructions. The result was expressed as pg/mL of CINC-1 normalized by total protein content (μg/mL), and given as pg/μg.

#### Molecular biology analysis of lung and diaphragm tissue

RT-PCR was performed to measure biological markers associated with inflammation (interleukin [IL]-6, cytokine-induced neutrophil chemoattractant [CINC-1]), alveolar stretch (amphiregulin), and fibrogenesis (type III procollagen [PC-III]) in lung tissue, as well as a marker of inflammation (tumour necrosis factor [TNF]-α) in diaphragm tissue. The primer sequences are listed in S1 Table. Central slices of the right lung or diaphragm were cut, collected in cryotubes, flash-frozen by immersion in liquid nitrogen, and stored at −80°C. Total RNA was extracted from frozen lung tissues using the RNeasy Plus Mini Kit (Qiagen, Hilden, Germany), following the manufacturer’s recommendations. The RNA concentration was measured by spectrophotometry in a Nanodrop ND-2000 system. First-strand cDNA was synthesized from total RNA using a Quantitec reverse transcription kit (Qiagen, Hilden, Germany). Relative mRNA concentrations were measured with a SYBR green detection system using ABI 7500 real-time polymerase chain reaction (Applied Biosystems, Foster City, CA, USA). Samples were measured in triplicate. For each sample, the expression of each gene was normalized to that of the housekeeping gene *36B4* (acidic ribosomal phosphoprotein P0) [26] and expressed as fold change relative to NV, using the 2^-ΔΔCt^ method, where ΔCt = Ct (reference gene)–Ct (target gene). All analyses were performed by one of the authors (C.L.B.), who was blinded to group assignment.

### Statistical analysis

The sample size was judiciously calculated to minimize the use of animals. A sample of 7 animals per group would provide the appropriate power (1-β = 0.8) to identify significant (α = 0.05) differences in volume fractions of the lung occupied by collapsed pulmonary areas between BIVENT-100 and BIVENT-50, since those groups showed distinct inspiratory efforts in previous studies by our group [7], taking into account an effect size d = 1.82, a two-sided test, and a sample size ratio of 1 (G*Power 3.1.9.2, University of Düsseldorf, Germany). The primary outcome was the difference in pneumonia score, whereas the secondary outcomes were respiratory system mechanics, lung heterogeneity, blood CFU count, and expression of genes related to inflammation, alveolar stretch, and fibrogenesis. The Kolmogorov–Smirnov test with Lilliefors’ correction was used to assess normality of data, while the Levene median test was used to evaluate the homogeneity of variances. To compare P_0.1_ level between High-Effort and Low-Effort groups, Student’s t-test was used (p<0.05). To compare functional parameters over time, a mixed linear model based on a random intercept for each animal followed by Bonferroni’s test was used (p<0.05). For lung damage, airspace enlargement, blood CFU count, and molecular biology assays in lung tissue, a Kruskal–Wallis test followed by Dunn’s multiple comparisons was performed (p<0.05). The mixed linear models were run in IBM SPSS Statistics for Windows, Version 19.0 (IBM Corp., USA). All other tests were performed in GraphPad Prism v8.1.1 (GraphPad Software, La Jolla, CA, USA).

## Results

No significant differences among groups were observed in the volume of fluids required to keep mean arterial pressure at or above 70 mmHg. Oxygenation improved over time only in the PSV group, whereas PaCO_2_ increased and pHa decreased in all groups. At FINAL, no significant differences were observed in oxygenation, PaCO_2_, pHa, or HCO_3_^-^ among groups (Table 1).

**Table 1:** Blood gas analysis during mechanical ventilation.

	Groups	INITIAL	FINAL	Time effect	Group effect	Interaction
**PaO**_**2**_**/FiO**_**2**_ **(mmHg)**				p = 0.003	p = 0.949	p = 0.328
	**PSV**	370 ± 35	442 ± 70†			
	**BIVENT**_**Low-Effort**_	386 ± 71	422 ± 86			
	**BIVENT**_**High-Effort**_	382 ± 49	408 ± 52			
**pHa**				p = 0.011	p = 0.439	p = 0.543
	**PSV**	7.37 ± 0.04	7.34 ± 0.03			
	**BIVENT**_**Low-Effort**_	7.39 ± 0.02	7.35 ± 0.09			
	**BIVENT**_**High-Effort**_	7.42 ± 0.03	7.35 ± 0.06			
**PaCO**_**2**_ **(mmHg)**				p = 0.048	p = 0.787	p = 0.674
	**PSV**	39 ± 8	46 ± 7			
	**BIVENT**_**Low-Effort**_	39 ± 5	42 ± 8			
	**BIVENT**_**High-Effort**_	37 ± 3	43 ± 6			
**HCO**_**3**_ **(mmol/L)**				p = 0.734	p = 0.831	p = 0.189
	**PSV**	21.9 ± 3.8	24.0 ± 2.5			
	**BIVENT**_**Low-Effort**_	23.9 ± 3.1	22.7 ± 2.1			
	**BIVENT**_**High-Effort**_	24.5 ± 1.4	22.7 ± 1.9			

Blood gas analysis at INITIAL and FINAL. PSV: pressure-support ventilation with ΔP set to achieve a V_T_ of 6 mL/kg (n = 7); BIVENT_Low-Effort_: Biphasic positive airway pressure at 50 controlled breaths/min. Animals were allowed to breath either at high and low positive airway pressures, and anesthesia was modulated to keep low inspiratory effort (n = 7). BIVENT_High-Effort_: Biphasic positive airway pressure at 50 controlled breaths/min. Animals were allowed to breath either at high and low positive airway pressures, and anesthesia was modulated to keep high inspiratory effort (n = 7). Values represent mean ± standard deviation (SD). Comparisons were done using a mixed linear model based on a random intercept for each animal followed by Bonferroni’s test was used (p<0.05).

† vs INITIAL (p<0.05).

**Fig 2:**
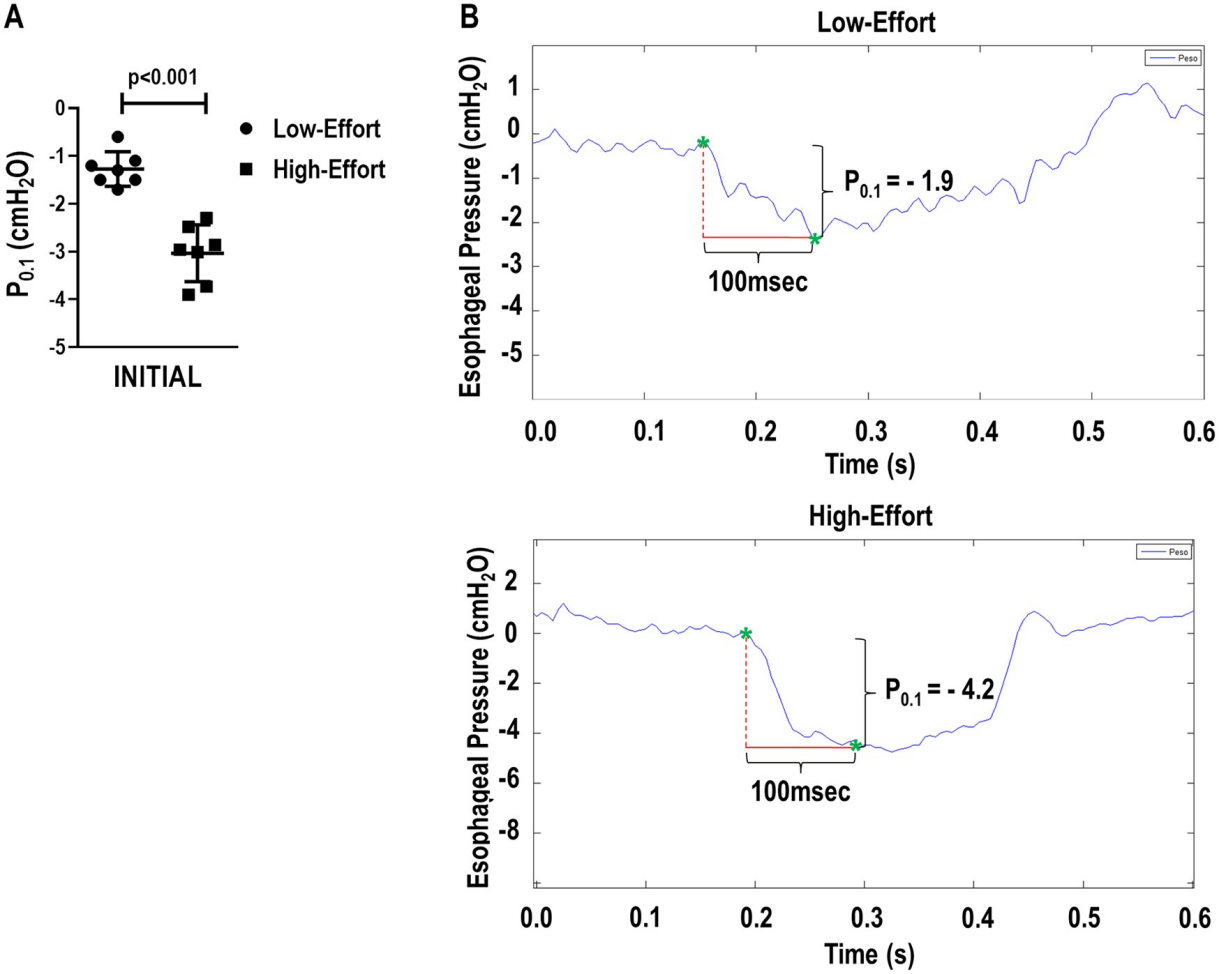
A. P_0.1_ values obtained at INITIAL in Low-Effort and High-Effort. B. Representative oesophageal pressure signals at INITIAL in Low-Effort and High-Effort. Low-Effort yielded a P_0.1_ value of -1.9 cmH_2_O, while High-effort yielded a P_0.1_ of -4.2 cmH_2_O. p<0.05 (Student’s t-test).

P_0.1_ was higher in BIVENT_High-Effort_ than BIVENT_Low-Effort_ and PSV (p = 0.002, and p<0.001, respectively) at INITIAL, and remained unaltered until the end of the experiments (Table 2). The representative P_0.1_ curves were different between BIVENT_High-Effort_ and BIVENT_Low-Effort_ (Fig 2). V_T_ did not differ among groups. At FINAL, Ppeak,_L_ was higher in BIVENT_High-Effort_ than BIVENT_Low-Effort_ and PSV (p = 0.015, and p = 0.011, respectively). RR and V′_E_ did not differ along time and between groups (Table 2).

**Table 2:** Respiratory variables during mechanical ventilation.

	Groups	INITIAL	FINAL	Time effect	Group effect	Interaction
**V**_**T**_ **(mL/kg)**				p = 0.289	p = 0.194	p = 0.621
	**PSV**	6.2 ± 0.3	6.2 ± 0.6			
	**BIVENT**_**Low-Effort**_	5.7 ± 0.4	6.0 ± 0.6			
	**BIVENT**_**High-Effort**_	5.8 ± 0.4	6.0 ± 0.4			
**RR (bpm)**				p = 0.455	p = 0.654	p = 0.739
	**PSV**	70 ± 11	70 ± 14			
	**BIVENT**_**Low-Effort**_	64 ± 3	70 ± 15			
	**BIVENT**_**High-Effort**_	72 ± 17	74 ± 16			
**V′**_**E**_ **(mL/min)**				p = 0.131	p = 0.601	p = 0.571
	**PSV**	140 ± 4	150 ± 5			
	**BIVENT**_**Low-Effort**_	143± 48	154 ± 52			
	**BIVENT**_**High-Effort**_	158 ± 24	189 ± 81			
**Ppeak,**_**L**_ **(cmH**_**2**_**O)**				p = 0.158	p = 0.013	p = 0.158
	**PSV**	13.0 ± 0.9	12.5 ± 1.6			
	**BIVENT**_**Low-Effort**_	12.0 ± 2.5	12.8 ± 1.5			
	**BIVENT**_**High-Effort**_	14.3 ± 1.9	16.1 ± 1.9*#			
**P**_**0.1**_ **(cmH**_**2**_**O)**				p = 0.027	p<0.001	p = 0.168
	**PSV**	-1.1 ± 0.7	-0.9 ± 0.8			
	**BIVENT**_**Low-Effort**_	-1.7 ± 0.6	-1.9 ± 0.8			
	**BIVENT**_**High-Effort**_	-3.0 ± 0.7*#	-4.2 ± 2.5*#			

Respiratory variables at INITIAL and FINAL. PSV: pressure-support ventilation with ΔP set to achieve a V_T_ of 6 mL/kg (n = 7); BIVENT_Low-Effort_: Biphasic positive airway pressure at 50 controlled breaths/min. Animals were allowed to breath either at high and low positive airway pressures, and anesthesia was modulated to keep low inspiratory effort (n = 7). BIVENT_High-Effort_: Biphasic positive airway pressure at 50 controlled breaths/min. Animals were allowed to breath either at high and low positive airway pressures, and anesthesia was modulated to keep high inspiratory effort (n = 7). V_T_: tidal volume; RR: respiratory rate; V′_E_: minute ventilation; *Ppeak*,_*L*_: *transpulmonary peak pressure;* P_01_: esophageal pressure measured after 100ms the beginning of inspiratory effort. Values represent mean ± standard deviation (SD). Comparisons were done using a mixed linear model based on a random intercept for each animal followed by Bonferroni’s test was used (p<0.05).

*, *vs* PSV;

^#^, *vs* BIVENT_Low-Effort_.

**Fig 3:**
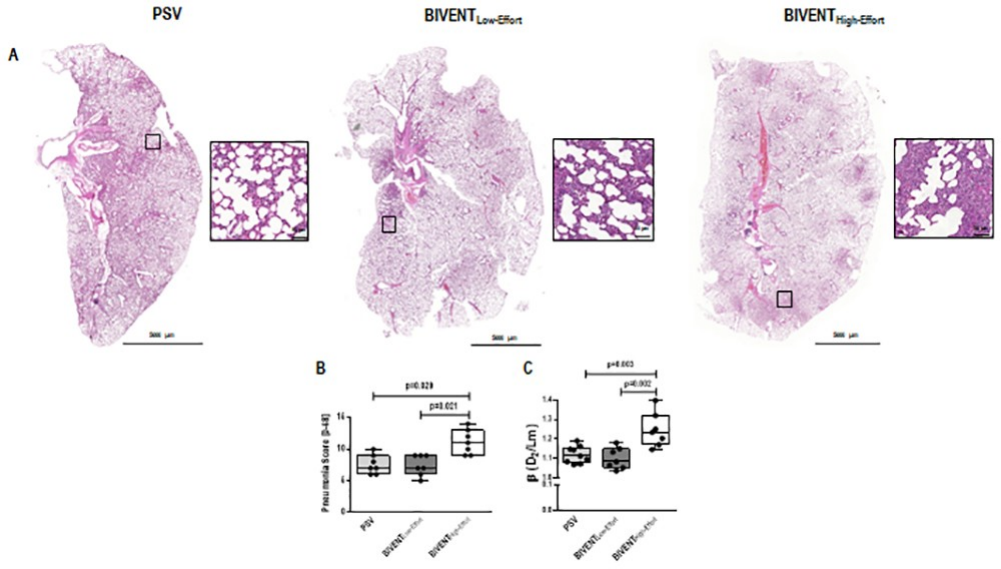
A. Representative scanned lung slices from PSV, BIVENT_Low-Effort_, and BIVENT_High-Effort_ groups. Inset: respective area selected from the whole lung. Bars at 50 μm. B. Pneumonia score [0–32] from PSV, BIVENT_Low-Effort_, and BIVENT_High-Effort_ groups. No difference was observed between PSV and BIVENT_Low-Effort_ (p = 0.999). C. Heterogeneity score (β) from PSV, BIVENT_Low-Effort_, and BIVENT_High-Effort_ groups. No difference was observed between PSV *vs* BIVENT_Low-Effort_ (p = 0.528). Box plots represent the median and interquartile range. Comparisons were done by Kruskal–Wallis test followed by Dunn’s multiple comparisons (p<0.05).

**Fig 4:**
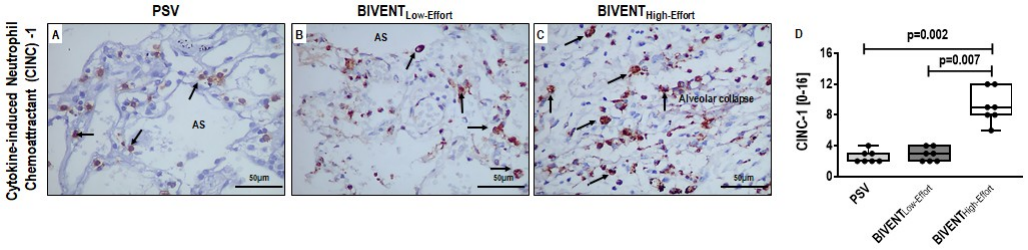
Representative photographs of immunohistochemistry of serial lung sections for CINC-1 (brown staining). In BIVENT_High-Effort_, intense CINC-1 expression was detected within a dense area of inflammation (panel C; arrows). Light staining for CINC-1 was observed in PSV group lung tissue (panel A; arrows). The middle panel shows moderate staining for CINC-1 in BIVENT_Low-Effort_ (panel B; arrows). AS: alveolar space. No difference in CINC-1 expression in lung tissue was observed between PSV and BIVENT_Low-Effort_ (p = 0.999). Box plots represent the median and interquartile range (panel D). Comparisons were done by the Kruskal–Wallis test followed by Dunn’s multiple comparisons (p<0.05).

**Fig 5:**
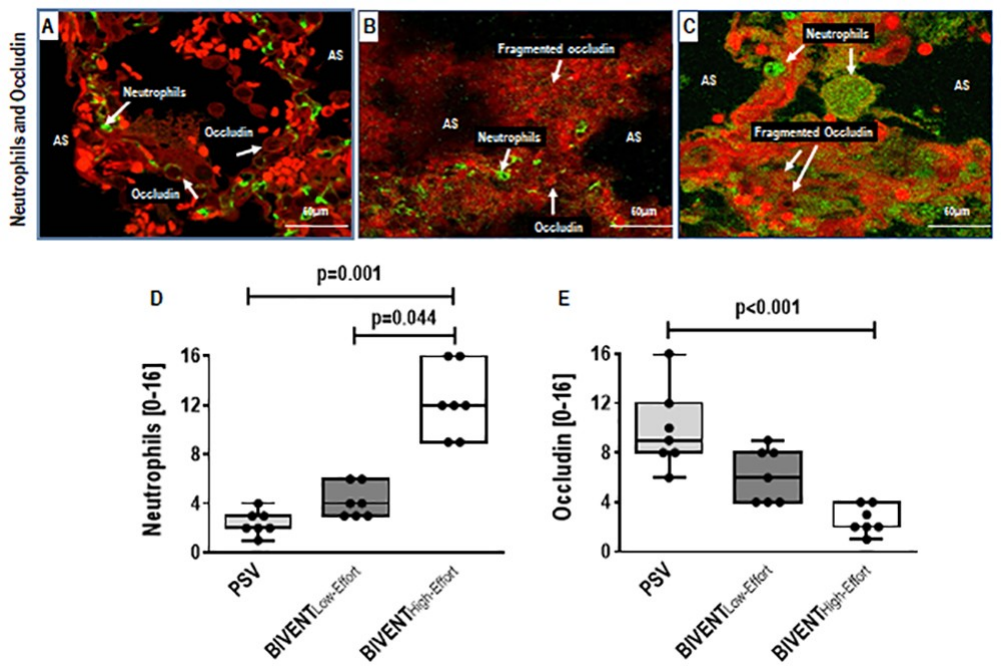
Double immunostaining of lung tissue for green-fluorescent neutrophils (GFN) and red-fluorescent occludin (RFO) from PSV, BIVENT_Low-Effort_, and BIVENT_High-Effort_ animals, visualized under confocal microscopy. Increased GFN cell counts (panel C; arrows) was seen in BIVENT_High-Effort_ compared with PSV (panel A; arrows) and BIVENT_Low-Effort_ (panel B; arrows) lungs. In contrast, increased RFO cell counts (panel A; arrows) were noted in PSV compared to both BIVENT_Low-Effort_ and BIVENT_High-Effort_. Note the fragmented occludin in both BIVENT groups. AS: alveolar space. No difference in neutrophil counts was observed between PSV and BIVENT_Low-Effort_ (p = 0.382). No differences in occludin were observed between PSV *vs* BIVENT_Low-Effort_ and between BIVENT_Low-Effort_ *vs* BIVENT_High-Effort_ (p = 0.350 and p = 0.078, respectively). Box plots represent the median and interquartile range (panels D and E). Comparisons were done by the Kruskal–Wallis test followed by Dunn’s multiple comparisons (p<0.05).

Pneumonia score was higher in BIVENT_High-Effort_ than BIVENT_Low-Effort_ and PSV, mainly due to increased oedema/haemorrhage (Fig 3, S2 Table). Accordingly, heterogeneity index was higher in BIVENT_High-Effort_ than BIVENT_Low-Effort_ and PSV (Fig 3). E-cadherin tissue expression did not differ among groups (median [interquartile range]: PSV, 27% [22% to 56%]; BIVENT_Low-Effort_, 39% [27% to 55%], BIVENT_High-Effort_: 32% [16% to 42%]; p = 0.547). CINC-1 protein expression in lung tissue was higher in BIVENT_High-Effort_ than BIVENT_Low-Effort_ and PSV (Fig 4). In addition, neutrophil cell counts were higher in BIVENT_High-Effort_ than BIVENT_Low-Effort_ and PSV (Fig 5D), while occludin expression was lower in BIVENT_High-Effort_ than PSV (Fig 5E). Occludin expression did not differ BIVENT_Low-Effort_ and BIVENT_High-Effort_.

**Fig 6:**
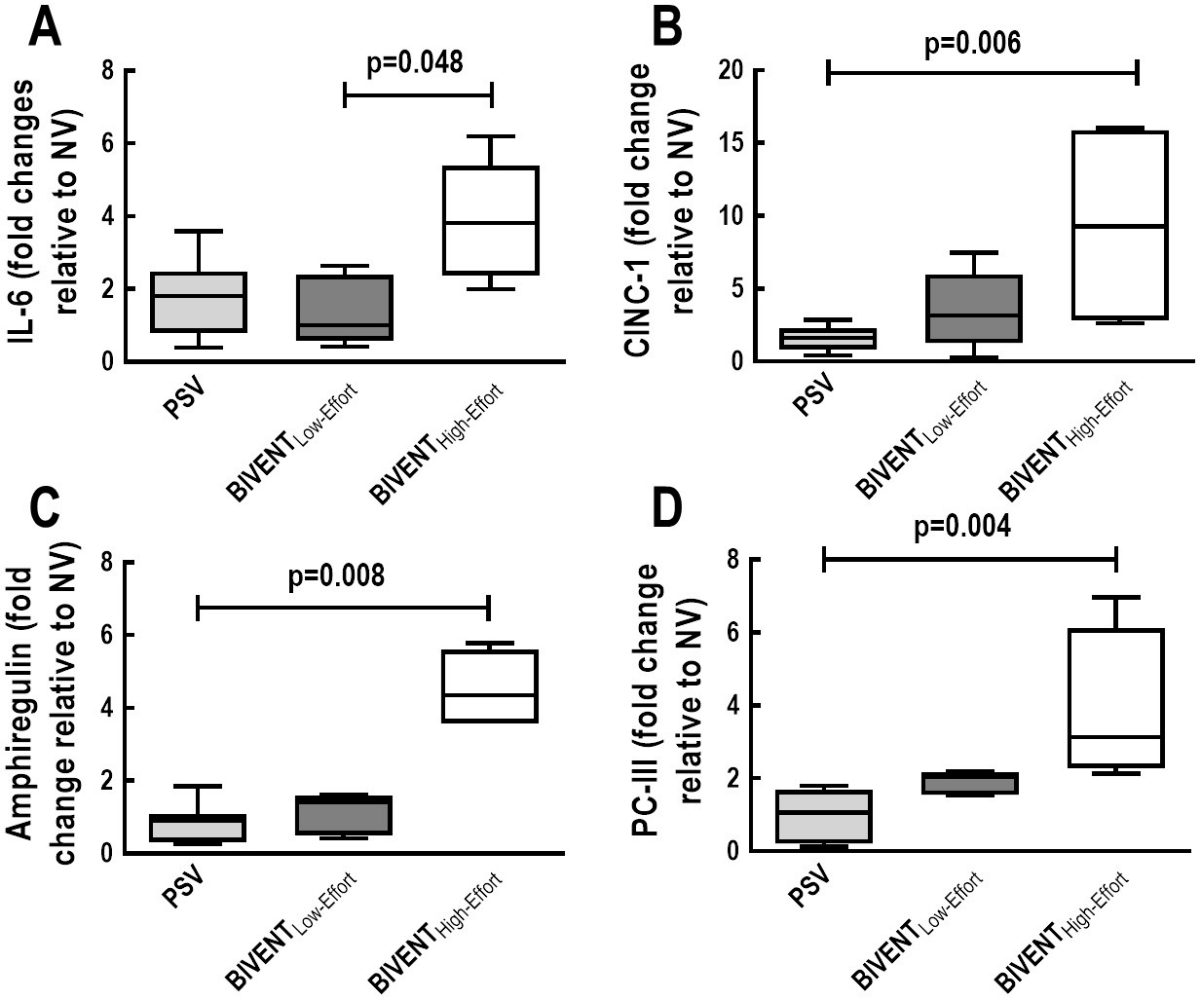
Real-time polymerase chain reaction analysis of biological markers measured in lung tissue for inflammation (A: interleukin [IL]-6; B: cytokine-induced neutrophil chemoattractant [CINC]-1), alveolar stretch (C: amphiregulin), and fibrogenesis (D: procollagen [PC]-III). Box plots represent the median and interquartile range. Relative gene expression was calculated as a ratio of the average gene expression levels compared with the reference gene (*36B4*) and expressed as fold change relative to respective NV. No difference was observed between PSV *vs* BIVENT_Low-Effort_ regarding IL-6, CINC-1, amphiregulin, or PC-III expressions (p = 0.996, p = 0.221, p = 0.999, p = 0.255, respectively). Comparisons were done by the Kruskal–Wallis test followed by Dunn’s multiple comparisons (p<0.05).

**Fig 7:**
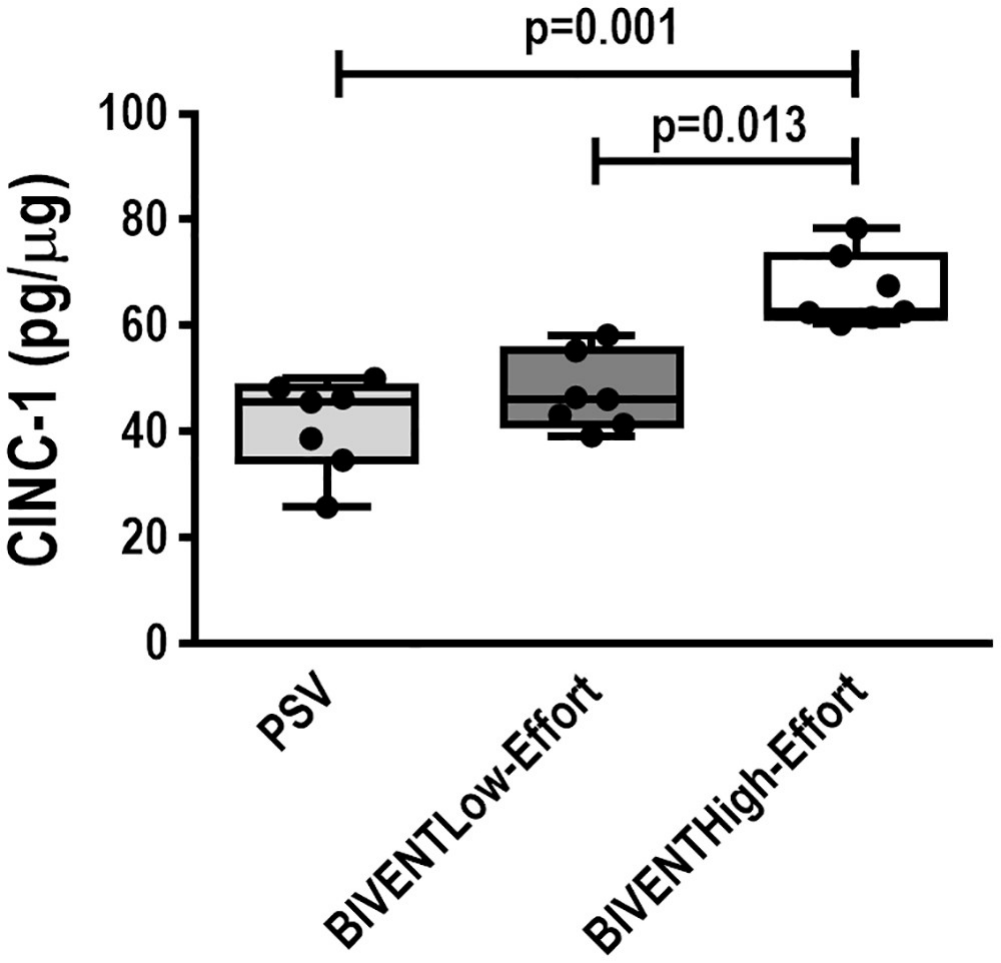
Protein levels of cytokine-induced neutrophil chemoattractant (CINC)-1 in lung tissue. Box plots represent the median and interquartile range in 7 animals/group. No difference in CINC-1 protein levels was observed between PSV and BIVENT_Low-Effort_ (p = 0.999). Comparisons among all groups were done by the Kruskal–Wallis test followed by Dunn’s test (p<0.05).

IL-6 and amphiregulin mRNA expression were higher in BIVENT_High-Effort_ than BIVENT_Low-Effort_ (Fig 6A and 6C). In addition, CINC-1, amphiregulin and PC-III gene expressions were higher in BIVENT_High-Effort_ compared to PSV (Fig 6B–6D). IL-6, CINC-1, amphiregulin, and PC-III expressions did not differ between BIVENT_Low-Effort_ and PSV (Fig 6A–6D). CINC-1 protein levels were higher in BIVENT_High-Effort_ than BIVENT_Low-Effort_ and PSV (Fig 7), but did not differ between BIVENT_Low-Effort_ and PSV.

**Fig 8:**
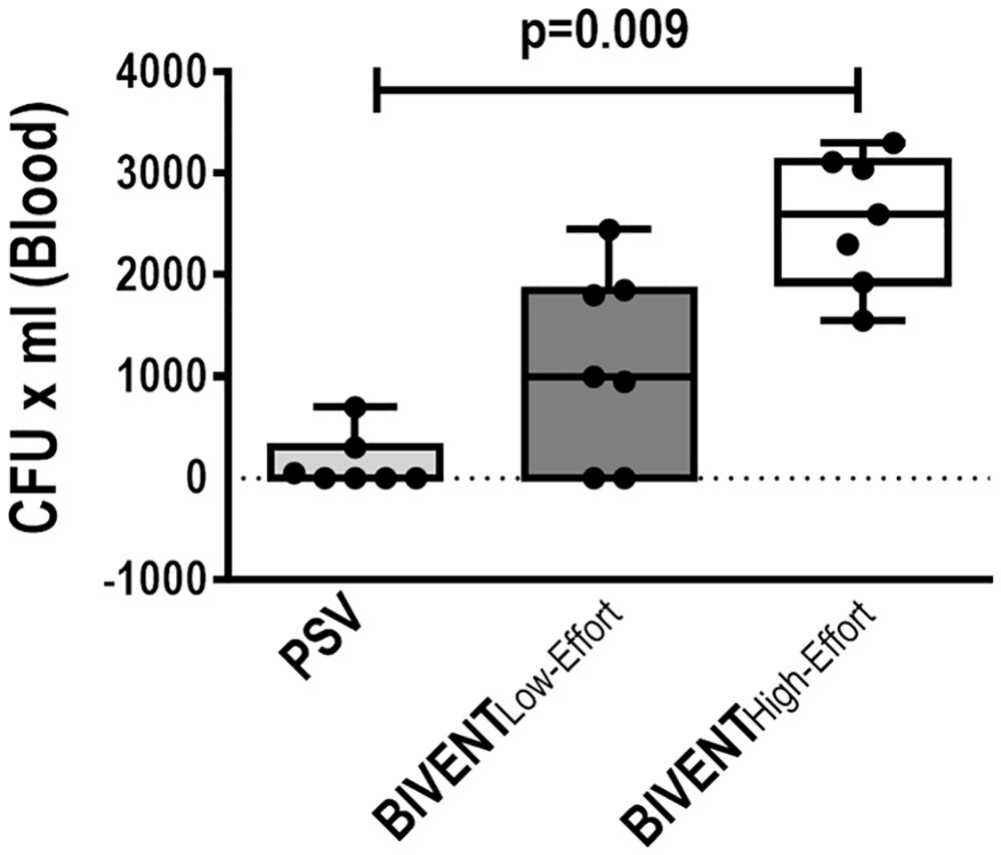
Blood bacterial counts. Lines represent the median and interquartile range of 7 animals in each group. The limit for detection was 50 CFU/ml. No difference was observed between PSV *vs* BIVENT_Low-Effort_ (p = 0.415) and BIVENT_Low-Effort_ *vs* BIVENT_High-Effort_ (p = 0.098). Comparisons were done by the Kruskal–Wallis test followed by Dunn’s multiple comparisons (p<0.05).

Blood CFU counts were higher in BIVENT_High-Effort_, but not in BIVENT_Low-Effort_, compared to PSV (Fig 8).

**Fig 9:**
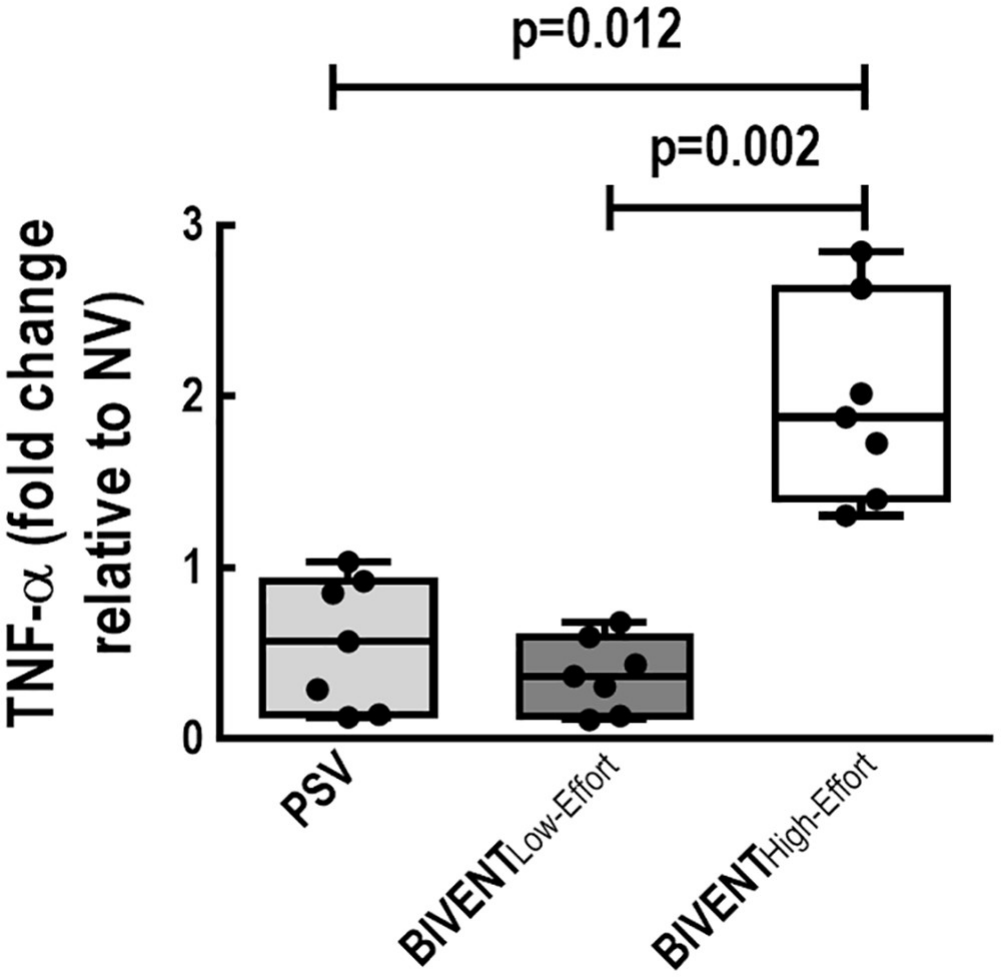
Real-time polymerase chain reaction analysis of tumour necrosis factor (TNF)-α in diaphragm tissue. Box plots represent the median and interquartile range. Relative gene expression was calculated as a ratio of the average gene expression levels compared with the reference gene (*36B4*) and expressed as fold change relative to respective NV. No difference was observed between PSV *vs* BIVENT_Low-Effort_ (p = 0.999) Comparisons were done by the Kruskal–Wallis test followed by Dunn’s multiple comparisons (p<0.05).

Diaphragm TNF-α mRNA expression was higher in BIVENT_High-Effort_ compared to BIVENT_Low-Effort_ and PSV (Fig 9); however, no difference was observed between BIVENT_Low-Effort_ and PSV.

## Discussion

In a model of *Pseudomonas aeruginosa*-induced pneumonia, rats mechanically ventilated with the same protective V_T_, we found that: 1) BIVENT_High-Effort_ was associated with higher transpulmonary peak pressure and histological damage score compared to BIVENT_Low-Effort_ and PSV; 2) BIVENT_High-Effort_ was associated with increased CINC-1 protein expression both in lung tissue and homogenate, while reduced occludin expression was detected in immunofluorescence analysis; 3) BIVENT_High-Effort_ increased IL-6 gene expression compared to BIVENT_Low-Effort_, as well as CINC-1, amphiregulin, and PC-III gene expressions in lung tissue compared to PSV; and 4) TNF-α gene expression in diaphragm tissue as well as blood bacterial counts were higher in BIVENT_High-Effort_ than BIVENT_Low-Effort_. BIVENT_Low-Effort_ and PSV, which also exhibited low inspiratory effort, were associated with similar mechanical, histological, and molecular behaviour. This suggests that, once a similar low degree of inspiratory effort is achieved, the mode of partial support ventilation does not affect VILI differently. On the contrary, BIVENT with high effort led to VILI in this model of pneumonia. In addition, P_0.1_ may be a feasible and easy tool to monitor and optimize the degree of inspiratory effort during assisted ventilation and thus minimize VILI.

*Pseudomonas aeruginosa* is a common cause of hospital-acquired pneumonia [1]. The present study used a rat model of *Pseudomonas aeruginosa*-induced pneumonia in which the CFU dosage was adjusted to result in blood bacteria with no mortality [16]. To date, there has been few research evaluating ventilatory strategies in pneumonia; current strategies are based on ARDS studies [27]. In preclinical studies in pneumonia, protective controlled mechanical ventilation (V_T_ = 6 ml/kg and PEEP = 10 cmH_2_O) was shown to reduce pulmonary bacterial burden in lung tissue [28], whereas different patterns of recruitment manoeuvres were responsible for moving mucus toward distal airways [29]. Both of these studies were done under controlled mechanical ventilation without muscle activity. The present study is the first to evaluate the impact of partial ventilatory support modes, at different inspiratory efforts, on lung and diaphragm damage in a pneumonia model. In this line, BIVENT was used since it is a partial support mode that employs pressure-controlled, time-cycled ventilation set at two levels of continuous positive airway pressure with unrestricted spontaneous breathing at any time of respiratory cycling [7]. In order to test the hypothesis regarding the role of different inspiratory efforts (low and high), anaesthesia was titrated by modulating ketamine and midazolam infusion rates, and P_0.1_ was kept constant throughout the experiment. To compare the effects of BIVENT with low and high effort, PSV, a method of spontaneous ventilation employed widely in clinical practice, was used [30]. During assisted ventilatory modes, protective V_T_ = 6 ml/kg was always applied.

### BIVENT_High-Effort_ *vs* BIVENT_Low-Effort_

As shown in Table 2, transpulmonary pressure was higher in BIVENT_High-Effort_ than BIVENT_Low-Effort_. Since a similar V_T_ was achieved between groups, we may infer that lung compliance was reduced in BIVENT_High-Effort_. This mechanical change was associated with a higher histological pneumonia score, mainly due to increased oedema/haemorrhage. In addition, biological markers associated with VILI were higher in BIVENT_High-Effort_ than BIVENT_Low-Effort_. Increased inspiratory effort can result in increased P_es_ swings and lung perfusion [31], thus increasing capillary leakage and oedema [32]. In lungs with bacterial pneumonia, normal alveolar areas coexist with adjacent consolidated areas (alveolar heterogeneity), which may lead to further lung damage when inspiratory effort is increased. Indeed, the heterogeneity score was higher with increased inspiratory effort. High inspiratory effort can cause a significant negative intrapleural pressure, leading to marked tensile stress [33]. Depending on the degree of tensile stress, alveolar unit overdistension and damage may occur even at the same V_T_ [11, 31, 34]. In the present study, the expression and protein level of CINC-1 in lung tissue homogenate were higher in BIVENT_High-Effort_ compared to BIVENT_Low-Effort_, which may result in increased neutrophil chemoattraction after *P*. *aeruginosa* infection [35]. Unfortunately, some ventilators do not detect this marked intrapleural pressure. As pointed out by Brochard et al [31], under partial ventilatory assistance, even though modest levels of positive airway pressure and V_T_ during inspiration are adjusted, one should not consider these patients to be protected. On the other hand, P_0.1_, which is a reasonable index of neuromuscular drive, can be measured in some ventilators and should be used much more widely at the bedside [31, 36]. The occurrence of spontaneous effort during the P_high_ period in BIVENT_High-Effort_ may increase transpulmonary pressure to dangerous levels.

### BIVENT *vs* PSV

BIVENT and PSV are different forms of partial support ventilation. PSV is a partial support mode which demands a inspiratory effort target for a given airway pressure range. The transition from inspiration to expiration occurs when the inspiratory flow has decayed to less than 25% of the peak inspiratory value (flow-cycled). On the other hand, BIVENT employs pressure control under partial ventilation (time-cycled), set at two levels of CPAP with unrestricted spontaneous breathing. Although there are fundamental differences regarding how BIVENT and PSV work, in the present study, we did not detect any difference between BIVENT_Low-effort_ and PSV, which denotes that the main cause of lung damage was not linked to type of partial ventilatory support, but rather was associated with the level of inspiratory effort. In this line, P_0.1_ was lower in PSV and BIVENT_Low-effort_ than in BIVENT_High-Effort_, in accordance with PSV values previously observed by our group in experimental ARDS [37, 38]. BIVENT_High-Effort_ was associated with higher pneumonia and heterogeneity scores compared to PSV. In addition, CINC-1 gene expression and protein levels, a marker of neutrophil migration into the lungs during pneumonia [39], was higher in BIVENT_High-Effort_ compared to PSV. The translocation of neutrophils may increase the gaps among endothelial and epithelial cells and, combined with overdistension, may predispose to fluid shift between capillaries towards the alveolar space. One way to infer the gaps among epithelial cells is to measure the expression of tight-junction proteins that promote epithelial integrity, such as occludin. Previous research has shown that the fragmentation of occludin followed by its reduction suggests epithelial integrity degradation by injurious mechanical ventilation [40]. Amphiregulin, a marker of alveolar stretch, has been found elevated in experimental influenza pneumonia [41], and was higher in BIVENT_High-Effort_ compared to PSV and BIVENT_Low-Effort_. Amphiregulin gene expression is mostly influenced by overdistension, and less so by inflammatory stimuli [42]. We hypothesize that the worse outcomes observed during partial ventilator support in pneumonia are related to the high tensile stress caused by high inspiratory effort, thus exceeding the plasto-elastic threshold of lung tissue [43] and contributing to bacterial translocation and distal organ damage [44].

Furthermore, high inspiratory effort resulted in increased gene expression of markers associated with diaphragm inflammation (TNF-α). The inappropriate use of partial ventilator support has been shown to injure not only the lung, but also the diaphragm [45]. One likely mechanism would be excessive concentric loading, during which vigorous contractions provoke high muscle tension, resulting in muscle inflammation, proteolysis, myofibrillar damage, and derangement of the sarcolemma [46]. Since the total time of mechanical ventilation was set to 1 hour, we chose to analyse the marker of inflammation which rises the fastest, TNF-α. We may further conclude that whether the mode of ventilation was flow- (PSV) or time-cycled (BIVENT) made no difference in terms of diaphragm injury, at least during low inspiratory effort.

### Possible clinical implications of study findings

In a prospective, observational, international multicentre cohort study of 13,751 ventilated ARDS patients, which had pneumonia as the major risk factor, spontaneous breathing was not associated with worse outcomes, and appeared to allow earlier weaning from the ventilator [14]. Nevertheless, it is well recognized that increased inspiratory effort has a negative impact on clinical outcome [11]. In the present study, we used BIVENT, which allows spontaneous ventilation at two levels of airway pressure. We also found that P_0.1_ may be a feasible parameter to monitor and optimize respiratory effort under partial ventilatory support modes [36].

### Limitations

Some limitations of this study must be noted. First, pneumonia was induced by intratracheal instillation of *Pseudomonas aeruginosa*; thus, our findings cannot be extrapolated to other types of pneumonia. Second, we only measured blood bacterial counts, not the distal organ contamination. Third, male animals were used to avoid the effects of female hormones on the expression of pro-inflammatory mediators [47], acknowledging the importance of sex differences in preclinical and basic studies [48]. Fourth, the ventilation period was limited to 1 h, since a longer period of time might result in haemodynamic instability and thus affect the expression of biological markers. One hour of mechanical ventilation was enough to observe molecular changes in key biological markers related to VILI and bacterial translocation from airspaces to the bloodstream. Further studies are required to evaluate whether the level of blood bacteria (CFU/mL) is associated with survival in mechanically ventilated rats during a long-term experiment. Fifth, PSV was done only at low inspiratory effort. Thus, we cannot ensure that, under high inspiratory effort, BIVENT and PSV would behave differently way from how they do under low inspiratory effort. A previous preclinical study found that PSV under intense work of breathing was not associated with worse outcomes [13].

## Conclusion

In the experimental model of *Pseudomonas aeruginosa*-induced pneumonia used herein, at the same protective V_T_, BIVENT under high inspiratory effort was associated with higher lung damage score and increased expression of genes associated with VILI and diaphragm inflammation, as well as increased translocation of bacteria into the bloodstream, compared to BIVENT and PSV (both with low inspiratory effort). Therefore, regardless of the mode of partial support ventilation (BIVENT or PSV), when low inspiratory effort was used, the lung and diaphragm remained protected and bacterial translocation was reduced. We also found that P_0.1_ might be a helpful parameter to optimize inspiratory effort during assisted ventilation.

## Supporting information

S1 Table Forward and reverse oligonucleotide sequences of target gene primers. (RTF)

S2 Table Pneumonia score. (DOCX)
