# Supplementary material for: Impact of positive biphasic pressure during low and high inspiratory efforts in Pseudomonas aeruginosa-induced pneumonia
Source: PLoS One. 2021 Feb 12;16(2):e0246891. doi: 10.1371/journal.pone.0246891 (PMC7880436; doi:10.1371/journal.pone.0246891)
Supplement: S1 Table — (RTF) [file pone.0246891.s001.rtf]

S1 Table: Forward and reverse oligonucleotide sequences of target gene primers

Gene	Primer	Primer sequences (5′-3′)	
Lung			
IL-6	Forward	CTC CGC AAG AGA CTT CCA G 	
	Reverse	CTC CTC TCC GGA CTT GTG A	
Amphiregulin	Forward	TTT CGC TGG CGC TCT CA 	
	Reverse	TTC CAA CCC AGC TGC ATA ATG	
CINC-1
	Forward	TGC ACC CAA ACC GAA GTC AT	
	Reverse	TTG TCA GAA GCC AGC GTT CAC	
PC-III	Forward	ACC TGG ACC ACA AGG ACA C	
	Reverse	TGG ACC CAT TTC ACC TTT C	
Diaphragm			
TNF-á	Forward	ACA AGC CCG TAG CCC ACG TC	
	Reverse	AGG AGC ACG TAG TCG GGG CA	
Housekeeping 			
36B4	Forward	AAT CCT GAG CGA TGT GCA G	
	Reverse	GCT GCC ATT GTC AAA CAC	

Primers used in experiments. IL-6, interleukin-6; CINC, cytokine-induced neutrophil chemoattractant; PC-III, type III procollagen; TNF-á, tumor necrosis factor-á; 36B4, acidic ribosomal phosphoprotein P0.
