# Supplementary material for: Impact of positive biphasic pressure during low and high inspiratory efforts in Pseudomonas aeruginosa-induced pneumonia
Source: PLoS One. 2021 Feb 12;16(2):e0246891. doi: 10.1371/journal.pone.0246891 (PMC7880436; doi:10.1371/journal.pone.0246891)
Supplement: S2 Table — (DOCX) [file pone.0246891.s002.docx]

|  | **PSV** | **BIVENT_Low-Effort_** | **BIVENT_High-Effort_** |
| --- | --- | --- | --- |
| Oedema / Haemorrhage [0-16] | 2.0 [2.0-3.0] | 2.0 [2.0-3.0] | 6.0 [4.0-6.0]*# |
| Septal Neutrophils [0-16] | 2.0 [2.0-3.0] | 2.0 [1.0-4.0] | 4.0 [2.5-4.0] |
| Vasculitis [0.16] | 2.0 [2.0-4.0] | 2.0 [2.0-4.0] | 2.0 [1.5-4.0] |
| Total [0-48] | 7.0 [6.0-9.0] | 7.0 [6.0-9.0] | 11.0 [9.5-13.0]*# |

**S2 Table:** Pneumonia Score

Pneumonia score representing injury from Oedema/haemorrhage, septal neutrophils, and vasculitis. PSV: pressure-support ventilation with ΔP set to achieve a V_T_ of 6 mL/kg (n=7); BIVENT_Low-Effort_: Biphasic positive airway pressure at 50 controlled breaths/min. Animals were allowed to breath either at high and low positive airway pressures, and anaesthesia was modulated to keep low inspiratory effort (n=7). BIVENT_High-Effort_: Biphasic positive airway pressure at 50 controlled breaths/min. Values are given as median (interquartile range) of 7 animals in each group. Kruskal–Wallis test followed by Dunn’s multiple comparisons was performed (p<0.05). * vs PSV; # vs BIVENT_Low-Effort_.
